# Lactate Like Fluconazole Reduces Ergosterol Content in the Plasma Membrane and Synergistically Kills *Candida albicans*

**DOI:** 10.3390/ijms22105219

**Published:** 2021-05-14

**Authors:** Jakub Suchodolski, Jakub Muraszko, Przemysław Bernat, Anna Krasowska

**Affiliations:** 1 Department of Biotransformation, Faculty of Biotechnology, University of Wrocław, 50-383 Wrocław, Poland; jakub.suchodolski@uwr.edu.pl (J.S.); jakub.muraszko@gmail.com (J.M.); 2 Department of Industrial Microbiology and Biotechnology, Faculty of Biology and Environmental Protection, University of Łódź, 90-237 Łódź, Poland; przemyslaw.bernat@biol.uni.lodz.pl

**Keywords:** *Candida albicans*, lactate, fluconazole, ergosterol, Cdr1

## Abstract

*Candida albicans* is an opportunistic pathogen that induces vulvovaginal candidiasis (VVC), among other diseases. In the vaginal environment, the source of carbon for *C. albicans* can be either lactic acid or its dissociated form, lactate. It has been shown that lactate, similar to the popular antifungal drug fluconazole (FLC), reduces the expression of the *ERG11* gene and hence the amount of ergosterol in the plasma membrane. The Cdr1 transporter that effluxes xenobiotics from *C. albicans* cells, including FLC, is delocalized from the plasma membrane to a vacuole under the influence of lactate. Despite the overexpression of the *CDR1* gene and the increased activity of Cdr1p, *C. albicans* is fourfold more sensitive to FLC in the presence of lactate than when glucose is the source of carbon. We propose synergistic effects of lactate and FLC in that they block Cdr1 activity by delocalization due to changes in the ergosterol content of the plasma membrane.

## 1. Introduction

*Candida* spp. are opportunistic pathogens that cause severe systemic infections in humans, such as vulvovaginal candidiasis (VVC) [1]. The main etiological species contributing to vaginal infections are *Candida albicans* (around 50%) and *Candida glabrata* (15–25%) [2,3]. Approximately 75% of women suffer from VVC at least once during their lifetime, and in many cases from recurrent VVC (RVVC) [4,5].

One of the factors defending the vagina against fungal infection is the presence of probiotic *Lactobacillus* spp. bacteria, which produce various short-chain aliphatic organic acids, such as lactic or acetic acid [6,7]. The concentration of acetic acid in the vaginal environment is low, ranging from 1–4 mM [8], while the lactic acid concentration in the vaginal milieu is around 110 mM [9]. Based on their results, Matsubara et al. [10] suggested that an antifungal effect occurs only after prolonged incubation with cultures of *Lactobacilli* (24 or 48 h) in which lactic acid could have accumulated in the medium at the right quantity. Other researchers have observed that the lactic acid concentration in the vaginal tract is too low to prevent the growth of all *Candida* spp. and that low pH plays a minor role in *Candida* spp. infections [11,12].

Despite these unfavorable facts, the presence of lactic acid increases the susceptibility of *C. albicans* to the antifungal compound fluconazole (FLC) [13]. Recently, Lourenco et al. [14] described a synergistic, reducing effect of lactic acid and FLC against *C. albicans*, but this effect was observed only in concentrations of lactic acid above 80 mM. Moreover, *C. albicans* growing on lactate is more resistant to amphotericin B (AmB), another antifungal drug [15]. The target for both antifungals is ergosterol that is present in the fungal membranes. FLC blocks the synthesis of ergosterol, while AmB binds to ergosterol and disturbs its functions in the membrane [16,17]. The reaction of *Candida* spp. to both antimycotics in the presence of lactic acid varies but indicates the role of ergosterol in these processes of resistance.

The mechanism of resistance of *C. albicans* to FLC consists mainly in the efflux of this compound by three membrane transporters, namely, Cdr1, Cdr2, and Mdr1. It was previously found that the type of carbon source affects the resistance of *C. albicans* to FLC [18]. Interestingly, the expressions of both *CDRS* and *MDR1* were decreased when the cells were grown on either glycerol or acetate compared with those grown on glucose [19]. Furthermore, Mira et al. [20] observed that the effect of undissociated organic acids on yeast cells is due to the perturbation of the plasma membrane (PM) structure, which can thus facilitate the introduction of the azole drug. Moreover, our research indicates a strong effect of changes to the lipid composition of the PM during resistance of *C. albicans* to azole compounds [21,22,23].

Acidic environments favor the existence of an undissociated form of lactic acid. Many previous studies have reported an absence of lactic acid toxicity against *C. albicans* at acidic pH [24,25], which could result from the ability to rapidly use lactic acid for metabolism [14]. As a Crabtree-negative organism, *C. albicans* can utilize glucose together with other carbon sources such as organic acids at the same time [26,27]. Therefore, when it uses lactic acid, it will prevent the acidification of its environment [12]. In a pH closer to neutral, a dissociated form of lactic acid, namely, lactate, prevails and can inhibit the growth of *C. albicans*. Despite these promising recent results, the influence of lactic acid and lactate on the tolerance of *Candida* spp. to azole compounds is still poorly understood.

In this work, we observed the increased sensitivity of *C. albicans* to FLC in the presence of lactate. We also explain the mechanism of inhibition of Cdr1 transporter activity by the lactate-dependent reduction of ergosterol in the PM. Hence, we propose that the mechanism of influence of lactate on the activity of Cdr1 transporters is based on our previous observations regarding ergosterol’s impact on membrane transporters [21].

## 2. Results

### 2.1. Lactate Affects the Resistance of C. albicans to Fluconazole Depending on Ergosterol—The First Observations

Both lactic acid and lactate can interact with *Candida* spp. depending on the pH of the vaginal environment [12]. Information regarding the mechanism by which lactate exerts its effects on *C. albicans* cells is scarce; hence we decided to investigate the susceptibility of *C. albicans* to FLC. This compound is effluxed from cells by PM *CDRS* transporters, mainly Cdr1 or Cdr2, in an auxiliary manner [28]. Our results show that when lactate is the sole carbon source, both the parental and mutant strains that have either one or both *CDRS* transporters removed are fourfold more sensitive to FLC than when they grow in the presence of glucose (Table 1). The absence of a difference in the sensitivity of *C. albicans* to FLC between the parental strain and the mutant with deletion in the *CDR2* gene indicates activity of only Cdr1p under the conditions utilized.

The use of brefeldin A and fluphenazine, which are substrates of Cdr1, did not induce a higher sensitivity of *C. albicans* in the presence of lactate compared with glucose as a carbon source (Table 1).

Brefeldin A is an inhibitor of LDH-mediated cholesterol efflux [29], whereas fluphenazine is a calmodulin antagonist [21]. Both compounds do not interact with the cell membrane or ergosterol. Based on the obtained results, we assumed that lactate could affect *C. albicans* synergistically with FLC by altering the amount of ergosterol in the PM and, for this reason, reduce the activity of Cdr1p. We decided to test the above hypothesis by carrying out further experiments.

### 2.2. Cdr1 Transporter Activity and CDR1 Gene Expression Are More Efficiently Upregulated in the Presence Lactate Compared with Glucose

**Figure 1:**
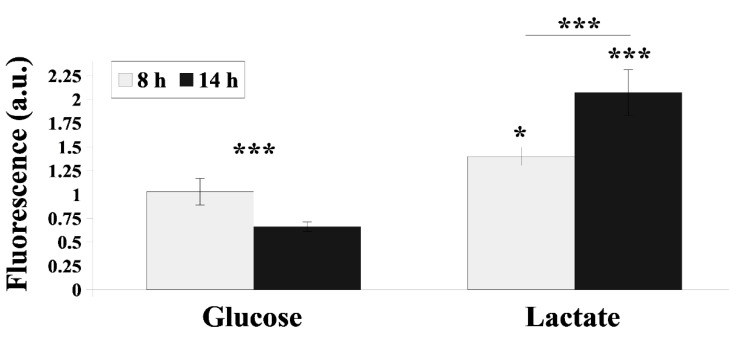
Cdr1p-dependent rhodamine 6G (R6G) efflux in *C. albicans* CAF2-1 strain during growth (8 and 14 h) in YPD (glucose) and YPL (lactate) media. The data are presented as normalized (=1 for 8 h YPD-grown strain) fluorescence intensity of extracellular R6G (mean ± SD, *n* = 3). Statistical analysis compared data during different growth phases (above lines) or compared YPD- and YPL-grown strains (above bars) (*, *p* < 0.05; ***, *p* < 0.001).

Cdr transporters are constitutively produced PM proteins that efflux xenobiotics from *C. albicans* cells, hence protecting them from death. Cdr1p activity varies depending on the growth phase of *C. albicans* and the presence of different compounds in the environment [21]. In the presence of lactate, the activity of Cdr1 was about 0.8- and 3-fold higher than when glucose was present, depending on the growth phase (Figure 1), and the reverse was also observed when lactate was the sole carbon source. In the late logarithmic phase of *C. albicans* growth with glucose-only media, Cdr1p activity decreased, while on lactate its activity significantly increased (Figure 1).

**Figure 2:**
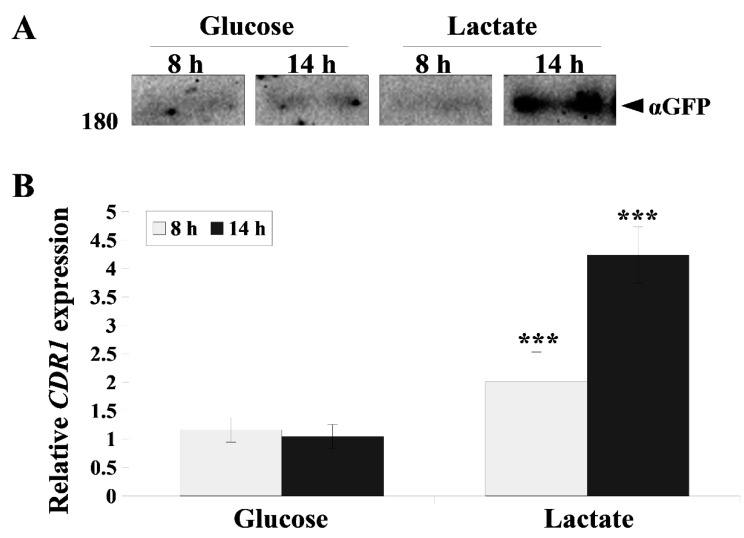
(**A**) Immunoblot analysis of Cdr1p levels in *C. albicans* ASCa1 (*CDR1-GFP*) strain during growth (8 and 14 h) in YPD (glucose) or YPL (lactate). The samples were resolved using 6% SDS-PAGE and probed with an anti-*GFP* antibody. Ponceau S staining was used as the loading control. The experiment was a representative of three independent assays, and the presented conditions were resolved in the same gel and cut out into separate lines (Figure S1 in Supplementary Materials). (**B**) Relative *CDR1* gene expression in *C. albicans* during different growth phases (8 and 14 h) in YPD or YPL medium. Gene expression levels are represented as mean 2^−ΔΔCT^ values ± SD (*n* = 6) normalized to 1 for 8 h YPD-grown gene expression level. Statistical analysis compared YPD- and YPL-grown strains (*** *p* < 0.001).

During the growth of *C. albicans* in the presence of lactate, not only did the activity of Cdr1p increase, but the amount of this protein significantly increased when visualized by Western blot (Figure 2A). The expression of the *CDR1* gene encoding this transporter was also higher, especially following 14 h of *C. albicans* growth (Figure 2B).

### 2.3. Lactate Accelerates Delocalization of Cdr1 Transporter from PM into Vacuoles

**Figure 3:**
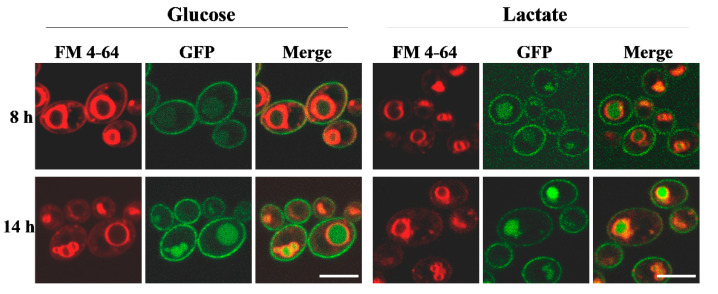
Confocal micrographs of vacuolar membrane staining (FM 4-64) and subcellular localization of *CDR1*-*GFP* protein in *C. albicans* ASCa1 (*CDR1-GFP*) strain during growth (8 and 14 h) in YPD (glucose) or YPL (lactate) medium. A merged image of the *GFP*-tagged protein and FM 4-64 staining is shown in the third and sixth columns. Scale bar = 5 μm.

To explain why there was a high sensitivity of *C. albicans* to FLC observed when grown in the presence of lactate despite an increase in Cdr1 expression and activity, we examined the localization of Cdr1p in the cells. It seems that in 8 h in a large amount, Cdr1p already delocalizes from the PM to vacuoles in the presence of lactate. When the source of carbon is glucose, Cdr1p mostly has the correct localization in the PM following 8 h of culture (Figure 3). In the late logarithmic phase (14 h), Cdr1p can be found in both the PM and vacuoles independent of the carbon source being utilized (Figure 3).

### 2.4. Lactate Inhibits Ergosterol Synthesis in C. albicans Cells

**Figure 4:**
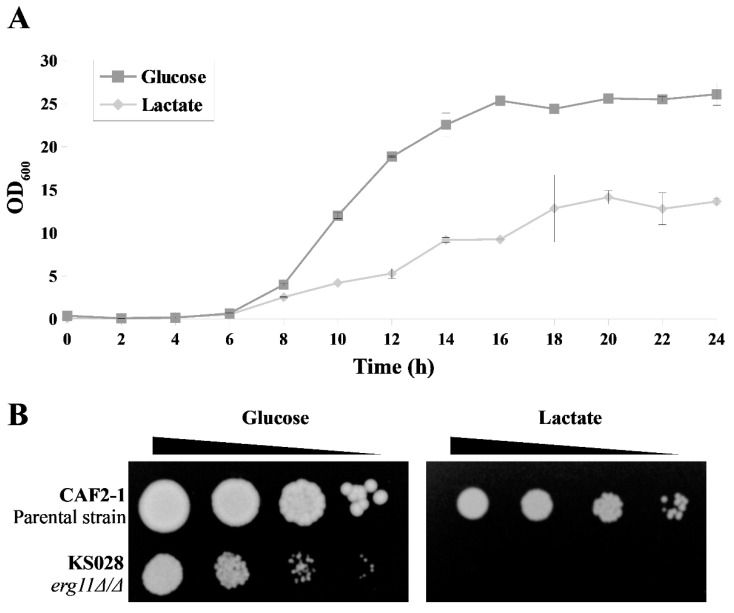
(**A**) Growth curves of *C. albicans* CAF2-1 strains in YPD (glucose) or YPL (lactate) medium (28 °C, 120 rpm), represented as OD_600_ (means ± SD, *n* = 4). (**B**) Growth phenotypes of *C. albicans* CAF2-1 (parental strain) and KS028 (*erg11Δ/Δ*) strains after 48 h incubation at 28 °C in YPD or YPL medium.

One of the reasons for the delocalization of Cdr1p from the PM to inside cells may be changes in the lipid composition of the membrane and mainly the loss of ergosterol [21]. The growth of *C. albicans* when lactate was the sole carbon source was significantly weaker than the growth in the presence of glucose (Figure 4A). When a mutant strain of *C. albicans* without ergosterol was cultured on lactate instead of glucose, we found that there was no growth present (Figure 4B).

**Figure 5:**
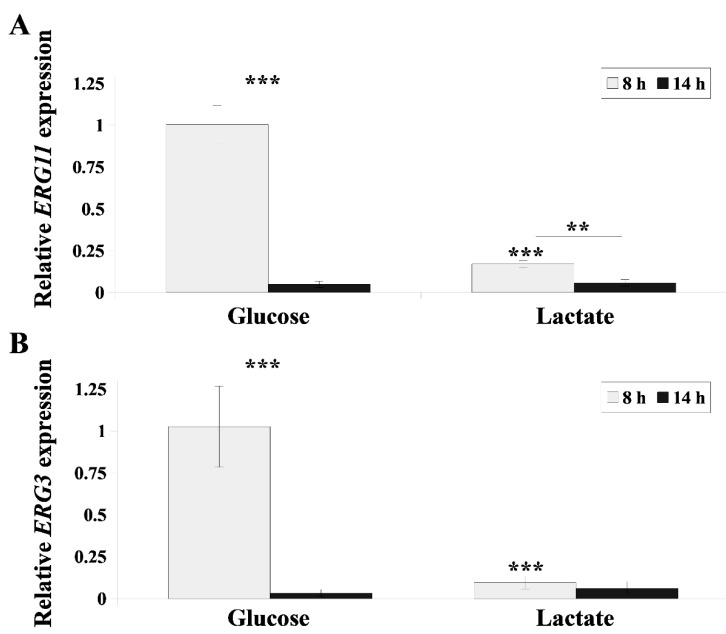
Relative *ERG11* (**A**) or *ERG3* (**B**) gene expression in *C. albicans* during different growth phases (8 h (grey) and 14 h (black)) in YPD or YPL medium. Gene expression levels as means of 2^−ΔΔCT^ values (*n* = 6) ± SD, normalized to 1 for 8 h YPD-grown gene expression level. Statistical analysis compared data during different growth phases (above lines) or compared YPD- and YPL-grown strains (above bars) (**, *p* < 0.01; ***, *p* < 0.001).

The intensity of the inhibition of ergosterol synthesis by FLC varies depending on the growth phase of *C. albicans* [30]. Thus, we investigated how high the expressions of the *ERG3* and *ERG11* genes are following 8 and 14 h *C. albicans* culture in the presence of lactate. Both genes are crucial in the sterol synthesis pathway in *C. albicans* [31]. Lactate inhibited the expression of *ERG11* by approximately 10-fold more than glucose following 8 h growth of *C. albicans*, while *ERG3* gene expression was similarly low in both phases of growth regardless of the carbon source (Figure 5).

The *ERG11* gene encodes lanosterol demethylase, which converts lanosterol into 4,4-dimethyl-5-cholesta-8,14,24-trien-3-ol, and the *ERG3* gene encodes C5 sterol desaturase, which converts episterol into 5,7,24(28)-ergostradienol in the ergosterol biosynthesis pathway [31]. As a result of the decrease in the expression of these genes, toxic sterols can accumulate in the membrane, which replace the missing ergosterol [32]. Deletion of both *ERG11* and *ERG3* genes leads to accumulation of eburicol and 14-α-methylfecosterol [33]. Analysis of sterols in *C. albicans* cells growing in the presence of lactate or glucose showed that in both 8 h and 14 h culture conditions, there was less (1.7-fold and 3.6-fold, respectively) ergosterol when cultured in lactate than in glucose (Table 2). For cells growing in the presence of lactate, 4-methylfecosterol was detected and in smaller amounts ergosta-5,7-dienol and 4,4-dimethylcholesta-8,24-dien-3b-ol, which were not found in cells cultured in the presence of glucose (Table 2). Lanosterol was at its highest level in cells grown on glucose as a source of carbon following 8 h of culture, while in other samples, lanosterol remained at a similar level (Table 2).

## 3. Discussion

Increasingly, research shows that the activities of antimicrobial drugs are strictly dependent on natural conditions that prevail in the environment of microbes [34,35]. Candidiasis treatment is no exception in this case, and it is important to take into consideration the effects of compounds that naturally occur in niches where *C. albicans* is present in the body. Recently, our research showed that fructose activates Mdr1 and Cdr1 transporters in *C. albicans* in response to the presence of FLC [18]. Furthermore, we demonstrated that capric acid produced by probiotic yeast *Saccharomyces boulardii* influences the susceptibility of *C. albicans* to FLC and AmB [23]. Lactate, a dissociated form of lactic acid, can occur in the vaginal environment when *C. albicans* rapidly absorbs lactic acid and the pH of the environment is raised [14]. This occurs when appropriate strains of *Lactobacillus* multiply in the microbiome of the vagina [12]. *Lactobacillus* and thus lactate or lactic acid may also be applied from the outside during treatment of candidiasis [36]. Our results presented herein indicate higher efficacy of candidiasis treatment when both lactate and FLC are used. We demonstrate that lactate lowers the expression of the *ERG11* gene of the ergosterol biosynthesis pathway in the early hours of *C. albicans* culture and induces incorrect localization of Cdr1, a transporter that removes FLC from cells (Figure 3 and Figure 5). According to our results, *C. albicans* increases *CDR1* gene expression and Cdr1p activity, as measured by R6G efflux, in the presence of lactate when compared with glucose, mostly in the late logarithmic phase of growth (Figure 1 and Figure 2). The time difference between inhibition of ergosterol synthesis and delocalization of Cdr1p from PM (8 h) and Cdr1p overproduction (14 h) appears too long for the cell to effectively defend itself against stress. This is also evidenced by accelerated growth of *C. albicans* on lactate compared with glucose (Figure 4A).

Analysis of sterols in the PM of cells growing in the presence of lactate showed a loss of the total amount of sterols in the membrane compared with cells growing on glucose. This was independent of the presence of new types of sterols (Table 2). In the study of Suchodolski et al. [21], the authors demonstrated that in the PM of the mutant *C. albicans* without ergosterol, there was an increase in the amount of sterols not normally present, particularly lanosterol, to compensate for the lack of ergosterol. In this work, we did not observe such compensation; hence the overall level of sterols was low in *C. albicans* that grew in the presence of lactate (Table 2). The *C. albicans* mutant with deletion of the *ERG11* gene did not grow on lactate (Figure 4B), and it can be assumed that lactate can also block cell defense after a complete loss of ergosterol.

We conclude that the synergistic effects of lactate and FLC are based on a similar mechanism of influence on *C. albicans*. Both compounds block the synthesis of ergosterol and cause delocalization of Cdr1p from the PM in the early hours of *C. albicans* growth. Cells cannot therefore efflux FLC; hence it has a fourfold lower MIC in this case (Table 1). The research of such interactions as we describe herein can help to improve the treatment of candidiasis and other diseases.

## 4. Materials and Methods

### 4.1. Reagents

The reagents used in this study were purchased from the following sources: 2-deoxy-D-glucose, fluconazole (FLC), rhodamine 6G (R6G), fluphenazine, β-mercaptoethanol (BME), ethylenediaminetetraacetic acid (EDTA), cholesterol, and BSTFA–TMCS (*n*,O-bis(trimethylsilyl) trifluoroacetamide/trimethylchlorosilane) (Sigma-Aldrich, Poznań, Poland). The following commercial antibodies were also used: a mouse monoclonal anti-*GFP* (Roche; distributor: Sigma-Aldrich, Poznań, Poland) and HRP-conjugated rabbit anti-mouse (GE Healthcare; distributor: Sigma-Aldrich, Poznań, Poland). D-glucose, bacteriological agar, zymolyase, D-sorbitol, brefeldin A (Bioshop; distributor: Lab Empire, Rzeszów, Poland), peptone, yeast extract (YE) (BD; distributor: Diag-Med, Warszawa, Poland), chloroform (CHCl_3_), methanol (MetOH), lactate (Chempur, Piekary Śląskie, Poland), FM 4-64 dye (Thermo Fisher, Warszawa, Poland). All reagents were analytical-grade compounds.

### 4.2. Strains and Growth Conditions

The *C. albicans* strains used in this study are listed in Table 3. CAF2-1, DSY448, DSY653, and DSY654 were gifts from Professor D. Sanglard (Lausanne, Switzerland). ASCa1 and KS028 were previously generated in our laboratory. Strains were maintained at 28 °C on YPD (1% YE, 1% peptone, 2% glucose) or YPL (1% YE, 1% peptone, 2% lactate) medium in a shaking incubator (120 rpm). Agar at a final concentration of 2% was used for solidifying the medium. For most of the experiments, cells were grown in 20 mL of YPD or YPL medium at 28 °C, shaking at 120 rpm with a starting A_600_ = 0.1. Cells were incubated until they reached the early (8 h) or late (14 h) logarithmic phase. Cells were then centrifuged at 4500 rpm for 5 min, washed twice with either phosphate-buffered saline (PBS) or 50 mM HEPES–NaOH buffer (pH 7.0), and resuspended in either PBS or HEPES–NaOH to the target A_600_.

### 4.3. MIC_50_ Determination

Experiments were performed in compliance with the Clinical and Laboratory Standards Institute (2008), 3rd ed. M27-A3, with modifications described previously [21]. Briefly, the MIC_50_ was determined by serial dilution of FLC, brefeldin A, or fluphenazine in YPD or YPL medium using sterile 96-well plates (Sarstedt, Stare Babice, Poland) and then inoculated with CAF2-1 cells at a final A_600_ of 0.01. After incubating at 28 °C for 24 h, A_600_ was again measured (ASYS UVM 340, Biogenet Józefów, Poland). The MIC_50_ was determined by normalizing the control A_600_ (without antimicrobial agents) as 100%.

### 4.4. Microscopic Studies

ASCa1 strain (*CDR1-GFP*), grown in YPD or YPL, was washed with PBS, concentrated by suspending the pellets in lower volume of PBS, and observed under a Leica SP8 LSM microscope (Leica Microsystems, Wetzlar, Germany). The staining of vacuolar membranes was performed with FM 4-64 dye as described previously [41].

### 4.5. Western Blot

Crude protein extract from ASCa1 strain was isolated as previously described [21,42]. Electrophoretic separation and transfer of Cdr1p-*GFP* was also performed as previously described [42]. For detection, a monoclonal mouse anti-*GFP* primary antibody was used, followed by an HRP-conjugated rabbit anti-mouse secondary antibody [18]. The remaining steps were performed as described [42].

### 4.6. Real-Time PCR

The isolation of RNA from *C. albicans* suspensions and cDNA synthesis was performed as previously described [43]. Briefly, total RNA from *C. albicans* cells was isolated using a Total RNA Mini kit provided by A&A Biotechnology (Gdynia, Poland). RNA was eluted with _dd_H_2_O. cDNA was obtained using a High-Capacity cDNA Reverse Transcription Kit (Thermo Fisher Scientific, Waltham, MA, USA). Then, samples were used as a matrix in quantitative PCR reaction. Gene expression levels were measured by quantitative PCR, which was performed with an iTaq Universal SYBR Green Supermix kit (Bio-Rad, Warszawa, Poland). Reactions were run using the StepOne Real-Time PCR System (Thermo Fisher Scientific, Waltham, MA, USA). Gene expression calculations were performed using the formula 2^−ΔΔCT^, with the *RDN18* gene as internal control, according to the protocol of Szczepaniak et al. [40]. The following gene-specific primers were used: RDN18F and RDN18R, ERG11F and ERG11R, ERG3F and ERG3R, CDR1F and CDR1R (Table 4).

### 4.7. Efflux Activity of Cdr1 Transporter

Suspensions of CAF2-1, in 25 mL HEPES–NaOH (A_600_ = 1.0), were grown in YPD or YPL medium with 2-deoxy-D-glucose and stained with R6G as previously described [18]. In each of the conditions, the R6G uptake was estimated to be ≥95%. Fluorescence intensities (FI) were collected 15 min after the initiation of the R6G efflux, and the efflux activity of CAF2-1 cells, grown in YPD, was normalized to 1.

### 4.8. Phenotype Assay

CAF2-1 and KS028 strain suspensions (PBS, A_600_ = 0.7, prepared from overnight YPD or YPL cultures) were serially diluted to 10^−3^. An amount of 2 µL was spotted onto either YPD or YPL agar, cultivated for 48 h at 28 °C. The plates were photographed using a FastGene^®^ B/G GelPic imaging box (Nippon Genetics, Tokyo, Japan).

### 4.9. PM Isolation and Sterol Analysis

PMs were isolated from suspensions of CAF2-1 grown in YPD or YPL medium (PBS, concentrated to A_600_ = 20) as previously described [21]. Briefly, cells were resuspended in lysis medium (1 M sorbitol, 0.1 M EDTA, 1% BME, 3 mg/mL zymolyase) and incubated at 37 °C for 30 min. Protoplasts were then washed with 1.2 M sorbitol, lysed with ice-cold _dd_H_2_O shock, and disrupted by sonication (5 s cycles for 2 min at 4 °C) using an ultrasonic processor (Sonics Vibra-Cell VCX 130, Sonics, Newtown, CT, USA). Cell lysate was centrifuged at 10,000 rpm at 4 °C for 10 min to remove unbroken material, and the supernatant was ultracentrifuged at 100,000 rpm at 4 °C for 60 min using a Micro Ultracentrifuge CS150FNX (Hitachi, Tokyo, Japan). The crude PM pellets were suspended in a chloroform–methanol solution (1:2 *v*/*v*). The chloroform layer was concentrated using nitrogen gas after vigorous stirring at 4 °C for 16 h. PM fractions were separated with BSTFA–TMCS, and sterol analysis was performed by gas chromatography–mass spectrometry (GC–MS) with cholesterol as an internal standard following the previously described protocol [21,44].

### 4.10. Statistical Analyses

Data represent the means ± standard deviation (± SD) from at least three independent replicates for each experiment. The exceptions were microscopic observations and Western blot analyses, which were performed in at least two independent replicates, of which representative images were included in the figures. Statistical significance was determined using Student’s *t*-test (binomial, unpaired), and a *p*-value of <0.05 was considered significant.

## Figures and Tables

**Table 1:** MIC_50_ (µg/mL) of FLC, brefeldin A, and fluphenazine against *C. albicans* cultured on YPD (glucose) or YPL (lactate) medium.

Strain *C. albicans*	Medium	Fluconazole	Brefeldin A	Fluphenazine
WT	YPD	2	16	125
	YPL	0.5	32	>250
*cdr1Δ*	YPD	0.25	4	125
	YPL	0.063	4	250
*cdr2Δ*	YPD	2	16	125
	YPL	0.5	32	>250
*cdr1Δcdr2Δ*	YPD	0.25	4	62.5
	YPL	0.063	4	62.5

**Table 2:** Sterols (µg/mg dry mass of isolated lipids, means ± SD, *n* = 3) in *C. albicans* CAF2-1 grown in YPD (glucose) or YPL (lactate) for 8 or 14 h. ND—not detected. Statistical analysis was performed in accordance with µg/mg values after 8 h of glucose culture (** *p* < 0.01; *** *p* < 0.001).

	Glucose		Lactate	
	8 h	14 h	8 h	14 h
Ergosterol	39.1 ± 2.3	65.2 ± 1.1 ***	22.9 ± 0.3 **	17.8 ± 4.8 ***
Lanosterol	8.5 ± 0.2	5.4 ± 0.6 **	5.9 ± 0.5 **	5.1 ± 0.8 **
4-methylfecosterol	ND	ND	3.9 ± 0.5	3.9 ± 0.2
Ergosta-5,7-dienol	ND	ND	1.1 ± 0.1	0.8 ± 0.2
4,4-dimethylcholesta-8,24-dien-3b-ol	ND	ND	1.3 ± 0.3	1.1 ± 0.1
Total sterols	47.6 ± 2.5	70.6 ± 1.7 ***	35.1 ± 1.7	28.7 ± 6.1 **

**Table 3:** *C. albicans* strains used in the study.

Strain	Genotype	Reference
CAF2-1	*ura3Δ::imm434/URA3*	[37]
DSY448	*ura3∆::imm434/ura3∆::imm434 * *cdr1∆::hisG/cdr1∆::hisG-URA3-hisG*	[38]
DSY653	*ura3∆::imm434/ura3∆::imm434 * *cdr2∆::hisG/cdr2∆::hisG-URA3-hisG*	[39]
DSY654	*ura3∆::imm434/ura3∆::imm434 * *cdr1∆::hisG/cdr1∆::hisG * *cdr2∆::hisG/cdr2∆::hisG-URA3-hisG*	[39]
ASCa1	*ura3Δ::imm434/ura3Δ::imm434 * *CDR1/CDR1-yEGFP-URA3*	[40]
KS028	*ura3Δ::imm434/ura3Δ::imm434 * *erg11Δ::SAT1-FLIP/erg11Δ::FRT*	[21]

**Table 4:** Primers used in the study.

Primer	Sequence 5′–3′
RDN18F	AGAAACGGCTACCACATCCAA
RDN18R	GGGCCCTGTATCGTTATTTATTGT
ERG11F	TTTGGTGGTGGTAGACATA
ERG11R	GAACTATAATCAGGGTCAGG
ERG3F	CCATCATGAATCATGACAGTCC
ERG3R	TGCTTCTCATGCTTTCCATC
CDR1F	TTTAGCCAGAACTTTCACTCATGATT
CDR1R	TATTTATTTCTTCATGTTCATATGGATTGA

## Data Availability

The data presented in this study are available on request from the corresponding author (A.K.).

## Supplementary Materials

The following are available online at https://www.mdpi.com/article/10.3390/ijms22105219/s1, Figure S1: Immunoblot analysis of Cdr1p levels in *C. albicans* ASCa1 (*CDR1-GFP*) strain during growth (8 and 14 h) in YPD (glucose) or YPL (lactate). The samples were resolved using 6% SDS-PAGE and probed with an anti-*GFP* antibody. Ponceau S staining was used as the loading control.
