# Supplementary material for: Lactate Like Fluconazole Reduces Ergosterol Content in the Plasma Membrane and Synergistically Kills Candida albicans
Source: Int J Mol Sci. 2021 May 14;22(10):5219. doi: 10.3390/ijms22105219 (PMC8156871; doi:10.3390/ijms22105219)
Supplement: Supplementary file 1 [file ijms-22-05219-s001.zip › ijms-1217987-supplementary.pdf]

International Journal of
Molecular Sciences

Supplementary materials

Initially we were experimenting with additional growth conditions, which were later excluded from our research. The western blot analysis presented in Figures 2 was cut into separate boxes to present only 8, 14 hr of growth on YPD (glucose) and YPL (lactate) media. In Figure S1 we present this immunoblot analysis with internal control (Ponceau S) included for western blot from Figures 2.

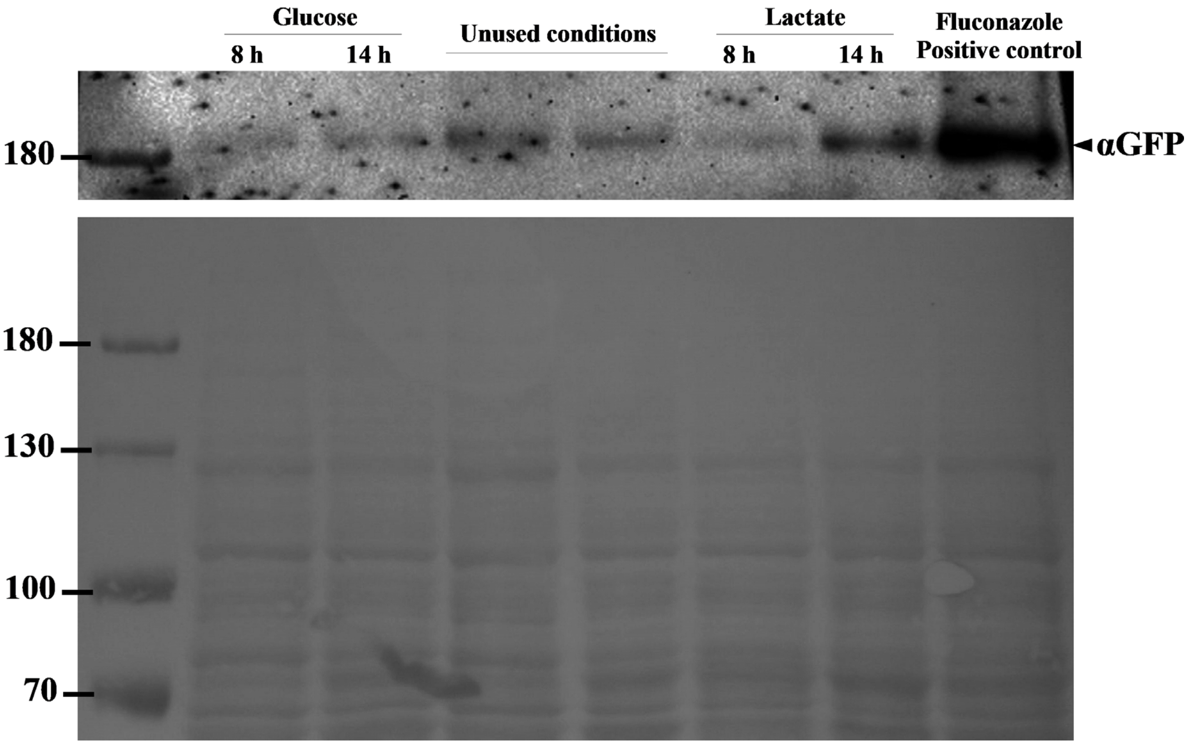

Figure S1. Immunoblot analysis of Cdr1p levels in *C. albicans* ASCa1 (*CDR1-GFP*) strain during growth (8 and 14 h) in YPD (glucose) or YPL (lactate). The samples were resolved using 6% SDS-PAGE and probed with an anti-GFP antibody. Ponceau S staining was used as the loading control.
